# Improving the computational accuracy of the dynamic electro-geometrical model using numerical solutions

**DOI:** 10.1038/s41598-022-09674-z

**Published:** 2022-04-06

**Authors:** Aderibigbe Israel Adekitan

**Affiliations:** Group for Lightning and Surge Protection, Technische Universität Ilmenau, Ilmenau, Germany

**Keywords:** Electrical and electronic engineering, Atmospheric science

## Abstract

The dynamic electro-geometrical model has been applied in various studies to investigate the probability of a lightning strike to parts of a structure. The numerical computation of the dynamic electro-geometrical model (DEGM) follows an iterative step by determining lightning strike points from above to a point on a structure of interest. This computation is often time-consuming and requires extensive computational resources. This study delves into the inner workings of DEGM striking distance computation. It highlights sources of computational numerical errors, such as the effect of the discretisation size. It proposes ways to eliminate such by using a conversion factor while also significantly reducing computation time from more than 14 h to approximately 6 min for a cuboid structure by eliminating ground surface points. The performance of the proposed improved DEGM (IDEGM) was investigated using a floating roof tank and a cuboid structure with a central air termination, and an interception efficiency of 61% was achieved. An alternative case using catenary wires with a total lightning interception efficiency of 99.1% was also implemented. The percentage strike probability for the cases considered shows a close approximation to published results, and this confirms the accuracy of the implemented model. The IDEGM has the benefit of generating results with a significantly reduced computation time of just a few minutes as compared to several hours in previous models.

## Introduction

Lightning occurs as a result of charges in the atmosphere, which produce electric fields that sustains the development of lightning leaders^1^. The flow path of lightning charges is random in nature as it searches for the optimal path in space^2^. This random nature makes designing a lightning protection system (LPS) challenging. LPS are installed on buildings and structures to prevent or significantly reduce the damage when lightning strikes such structures^3,4^. A building is not automatically protected by installing air terminations, except if such air terminations are adequately designed^5^ and positioned at high-risk points to ensure the interception of downward leaders by the streamers emitted by such rods before the lightning terminates on the protected structure. When there is only a small difference in height between the protected structure and the air termination, the position of the air termination becomes very critical^6^. Air terminations can be in the form of rods, tapes or catenary wires. Blunt tipped rods have been found to perform better than sharp ones as regards lightning interception^3^. Lightning current flows for a short duration of a few hundred microseconds, but the high energy transient current can cause damage if it flows through unintended objects. Intercepting a lightning strike by air terminations before it strikes the protected structure is just the first step in a three-step process. The lightning current must also flow safely through dedicated and well-designed down conductors on the structure. Ultimately, the current must flow safely to the earth through a low resistance grounding. Otherwise, lightning bypass to nearby objects, animals and humans may occur. Likewise, sensitive devices and electronics may be damaged if surge protective devices are not installed on power lines^7^ and signal lines entering the structure. The level of protection offered by a LPS is classified into four by the International Electrotechnical Commission (IEC) 62,305^8,9^.

The likelihood of a direct lightning strike to different parts of a structure is greatly influenced not only by the geographical location of the structure but also by the shape and the dimension of the structure itself, and tall structures have a higher risk of a direct lightning strike^6,10^. Lightning is a natural occurrence, and the scientific community has made efforts to understand its nature, even to the extent of predicting its occurrence and geolocation by lightning location systems (LLS) using sensors that monitor the electromagnetic fields associated with lightning^11,12^. Knowing lightning behaviour and the attachment process to a structure is important for developing models and guidelines for implementing adequate and efficient LPS for structures. If the position of air terminations on a structure is vital for its efficacy, then the question arises, how should such high-risk points be identified? The rolling sphere method, which uses an imaginary sphere rolled on the structure of interest, has been used over time to determine the placement of air terminations^13,14^. While the rolling sphere indicates likely strike points on a structure, it does not clarify differences in the level of exposure and therefore assumes that all points identified have the same level of risk or probability of a direct strike, which is not true. Also, the interception distances computed by the rolling sphere method have been described to be overestimated for grounded structures^15^.

The likelihood of a direct strike to various parts of a structure can be quantified in terms of probabilities, and this was applied to evolve the dynamic electro-geometrical model^16–18^. The numerical DEGM can be used to evaluate the likelihood of a direct strike to the meshed surface of a structure. The DEGM involves discretising the structure and surrounding space from which lightning can strike the structure into meshed points. The numerical implementation of DEGM is computer-intensive due to the number of iterations required. Modifications to the definition of the space point around a structure can be implemented. The modifications will help to reduce the computation time to several hours rather than days, as observed for some structures, even on a computer with good specifications^19^. The advantages of the DEGM are obvious in terms of its ability to quantify the likelihood of a direct strike to the various parts of a structure. The numerical DEGM has challenges in terms of computation time and associated numerical errors. Based on extensive works in this area, this study presents the various sources of numerical errors that have been identified, which impacts the accuracy of the DEGM results. In this work, novel concepts are proposed for eliminating identified sources of numerical errors towards improving numerical accuracy by applying a probability density function (PDF) to cumulative distribution function (CDF) conversion factor. The sources of numerical errors are first identified and discussed, and methods for improving the model accuracy and computation time are then proposed. The concepts developed were applied to a free-standing rod, a cuboid, a cuboid protected with air terminations, and a floating roof tank. Also, a method that removes the ground meshed points from the iterations is proposed to reduce the computation time from more than 14 h achieved by Adekitan and Rock^19^ to less than 6 min for cuboid structures and from 27.38 h to less than 30 min for a floating roof tank (FRT). The results show improved accuracy when compared with published results from alternative approaches. For the cuboid structure with a central air termination, the interception efficiency is 61% as compared to 99.91% using four catenary wires. This finding highlights the importance of accurate air termination design and positioning to ensure effectiveness.

## Numerical errors and slow computation factors in the dynamic electro-geometrical model

Two major factors which introduce numerical errors into DEGM simulation are discussed in this section, together with space point definition, a major factor that determines computation time.

### Discretisation size of surface and space points

**Figure 1:**
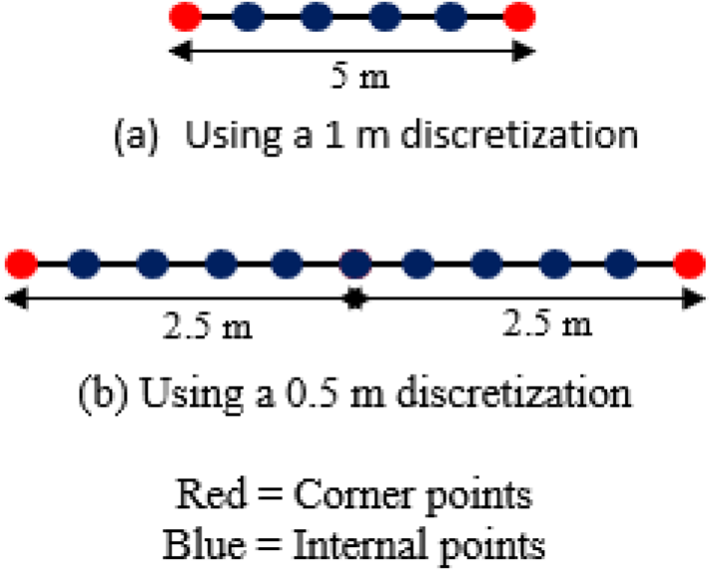
The effect of discretisation size on surface length.

Implementing the DEGM requires the discretisation of surfaces on the structure and surrounding space. The size of the discretisation impacts the accuracy of the DEGM model. The discretisation size affects the effective length of the surface as compared with the actual length. For example, for a surface length of 5 m on the side of a cuboid, as illustrated in Fig. 1a, a 1 m discretisation will produce 2 endpoints which can be referred to as corner points and 4 inner points. The computation for endpoints is different from that for the inner points. In an analytical computation, the probability modulated collection volume (PMCV) for a length L is equal to L multiplied by the PMCV for a meter, i.e., in this case, it is 5 × PMCV per unit. In the numerical model, because there are only 4 inner points, the resulting value is 4 × PMCV per unit. This implies that the value for 1 m is lost. If a discretisation size of 0.5 m is applied to the same 5 m surface length, the resulting analysis is shown in Fig. 1b, in which there are 2 endpoints and 9 inner points. The 9 inner points represent 4.5 m which means the value for 0.5 m is lost. The smaller the discretisation size, the better the accuracy, but the longer the computation time. There needs to be a trade-off between accuracy and computation time when selecting a discretisation size. A maximum discretisation size of 1 m × 1 m is recommended for square meshes.

### Discontinuity in the striking distance path due to vertical space layers

**Figure 2:**
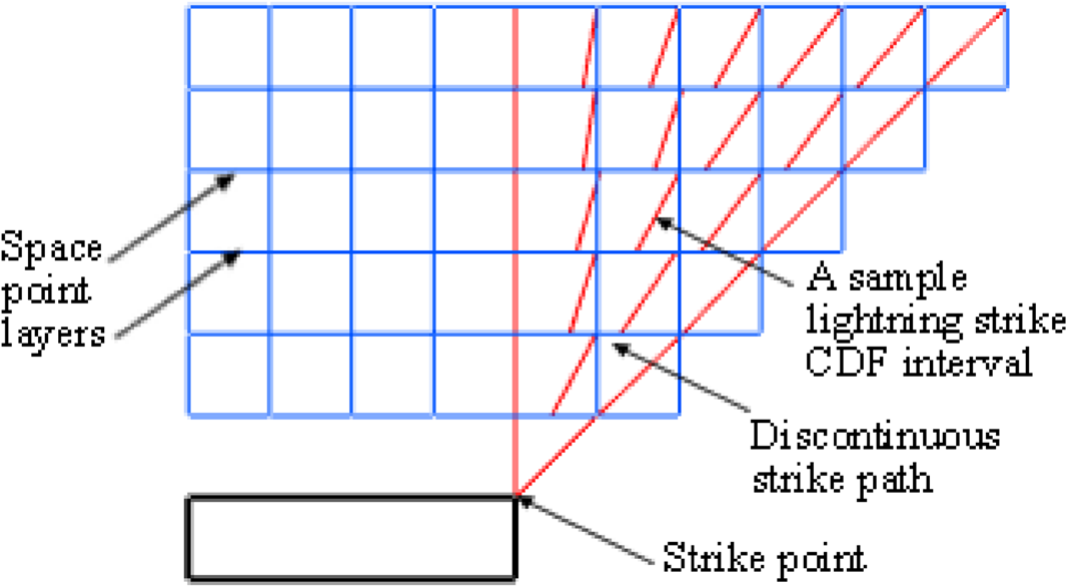
The discontinuous effect due to the vertical discretisation of space layers.

The space point layers are modelled at vertical intervals, e.g., at 10 m intervals above the top of the structure under analysis, as illustrated in Fig. 2. The shorter the interval, the more the computation time. For space points above the structure, the cumulative distribution function is computed upwards from the top of the structure to 300 m above it. At 300 m, the CDF is already 0.99 out of the total of 1. The CDF between any two points, one on the upper layer with distance r_2_ from a particular surface point on the structure and the other on the lower layer with distance r_1_, is computed with Eq. (1), where PDF is the striking distance (*r*) probability density function and Eq. (1) is written as

1 $$CDF_{{{\text{interval}}}} = \int_{{r_{1} }}^{{r_{2} }} {PDF(r)\;dr} .$$

The vertical layers of space point also create a form of discontinuity for the modelled striking distance path. The striking distance, by definition, is a straight line from the point of orientation in space to the strike point on a structure. For space points directly above the roof, the strike paths are vertically above each other such that it forms a straight line. For some other space points, a plot of the lines will show a discontinuity from one space point layer to another as it does not form a straight continuous slant line, and this is due to the vertical discretisation of space point layers, e.g., 10 m intervals and the horizontal discretisation, e.g., 1 m × 1 m, as illustrated in Fig. 2. This discontinuity along the striking distance will impact the accuracy of the cumulated CDF across each strike path.

### Excessive space points in the model beyond the collection volume

**Figure 3:**
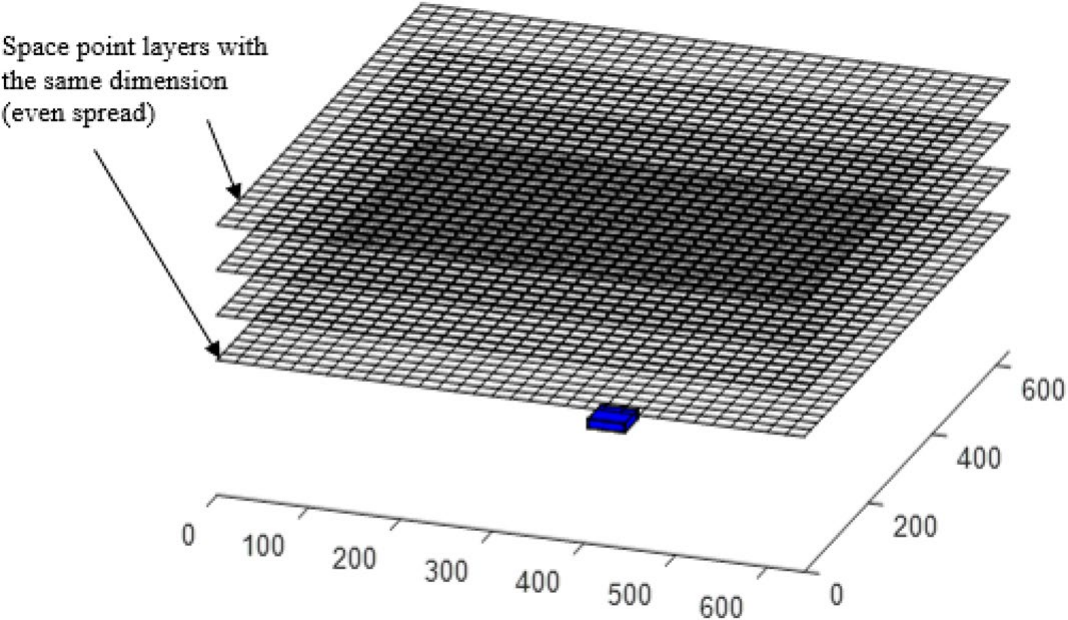
Space point layers evenly spread over the structure^19^.

The numerical approach to DEGM is particularly slow because of the number of space point iterations required to compute the solution. The use of evenly spread space points layers above the structure with the same maximum horizontal distance from each side of the cuboid, as shown in Fig. 3, includes a significant number of space points that are not within the collection volume of the structure and, as such, including them in the simulation only increases computation time as they do not affect the result. The evenly spanned space points can be modified to form linearly increasing space points. This approach can also be further compacted by using rectangular space points confined around the collection volume. This will significantly reduce the number of iterations to about 6% without impacting the accuracy of the cases evaluated^19^.

**Figure 4:**
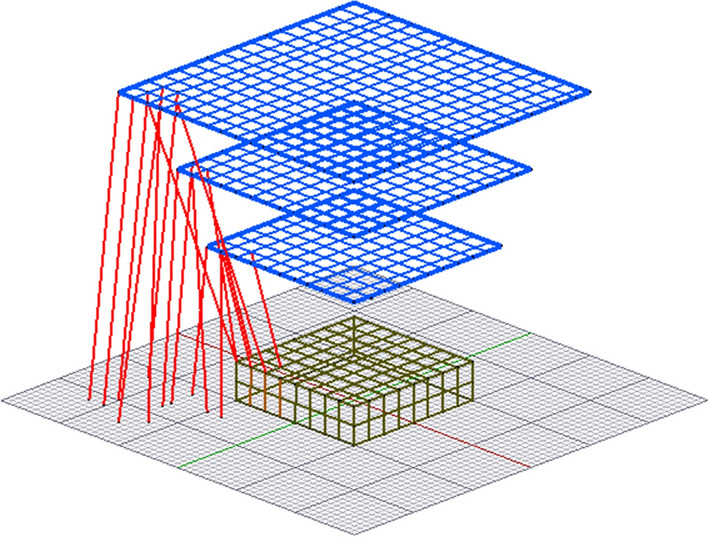
Superfluous space points with strikes to the ground together with needed space points with strikes to the structure at the corner area of the space point layers.

As illustrated in Fig. 4, the confined space layers still have some strikes that are closer to the ground than to the structure. These strikes are from space points at the corner edges of the rectangular space layers. Therefore, there is an opportunity here to refine further the definition of the space layers models to eliminate the superfluous space points, and by so doing, all the remaining space points will be completely within the collection volume of the structure. This implies that there is no need to identify and separate strikes to the ground from strikes to the structure. The discretised ground surfaces and associated iterations can be eliminated from the implementation of DEGM to offer a significant computation time advantage.

## Methodology

**Figure 5:**
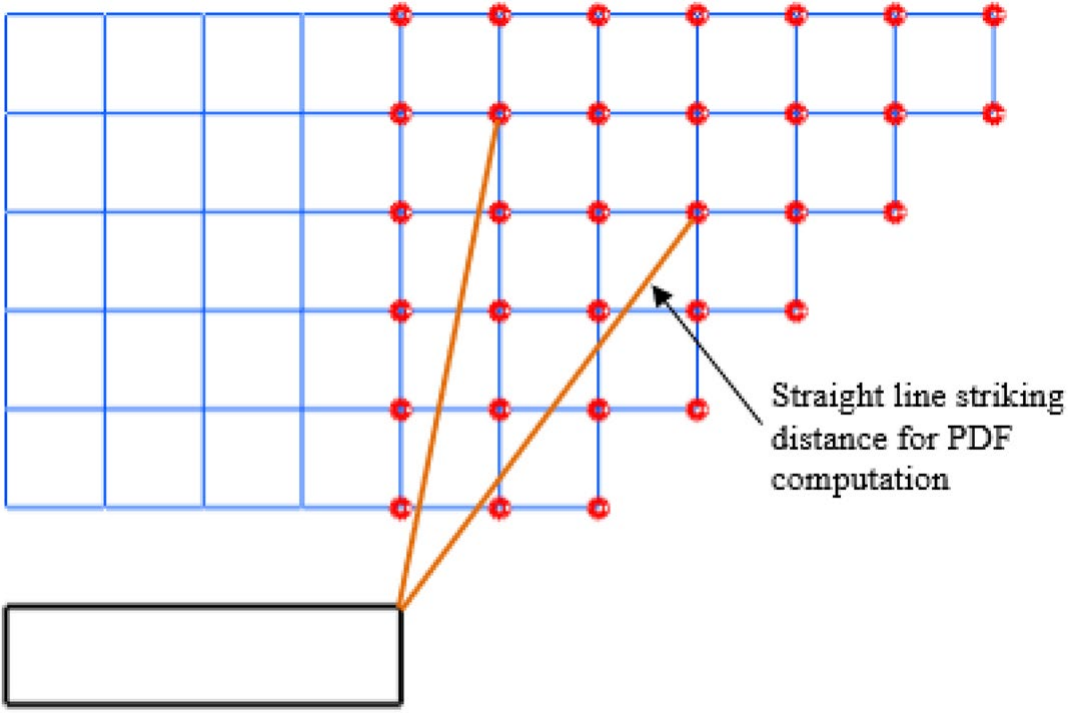
PDF-based pointwise computation of strike PMCV.

Modifications to the numerical DEGM implementations are hereby proposed in this section to improve the numerical solution’s computational accuracy. The proposed improved dynamic electro-geometrical model (IDEGM) uses concepts from the traditional implementation with modifications to improve performance. The accuracy of the computation of the probability modulated collection volume (PMCV) ultimately determines the accuracy of the DEGM. The use of CDF is a space point interval striking-distance based approach, and it is susceptible to path discontinuity. To improve on this concept, an approximation to the CDF by applying the PDF without integration across any space interval is proposed. Instead of an interval-based definition, a pointwise analysis is presented, as illustrated in Fig. 5. The PDF based on the striking distance between a space point and the surface point on the structure or the ground will be determined instead of the CDF. A conversion factor must be applied to convert this value to CDF. The computation of CDF on a straight line from 0 to 300 m is 0.9902. If a space level vertical interval of 10 m is applied, the PDF as defined in Eq. (2) is computed from 0 m at an interval of 10 m up to 300 m, and the summation is 0.0966. Based on this, a PDF to CDF conversion factor hereby referred to as K_P2C_ is proposed, and K_P2C_ = 10.25. Note that this factor is defined here for a vertical space point level interval of 10 m above the structure. If other spacing is applied, the factor must be recalculated. For most structures, except for very tall ones, space point to the side of the structure can be defined with a vertical interval of 1 m, and as such, the K_P2C_ will not be required for such points. For space points above the roof of the structure up to 300 m, using a space level interval of 10 m, K_P2C_ must be applied to convert the PDF to CDF for each space point.

The probability density function as a function of striking distance (*r*) is defined in Eq. (2) according to the definitions by IEC 62,305–1, Annex A^8^, where

2 $$PDF(r) = \frac{{10^{{\frac{ - 1}{{0.65}}}} }}{0.65}r^{{\frac{0.35}{{0.65}}}} \frac{1}{{\sigma \left( \frac{r}{10} \right)^{{\frac{1}{0.65}}} \sqrt {2\pi } }}e^{{\frac{{ - \left\{ {\ln \frac{{\left( \frac{r}{10} \right)^{{\frac{1}{0.65}}} }}{\mu }} \right\}^{2} }}{{2\sigma^{2} }}}} .$$

**Figure 6:**
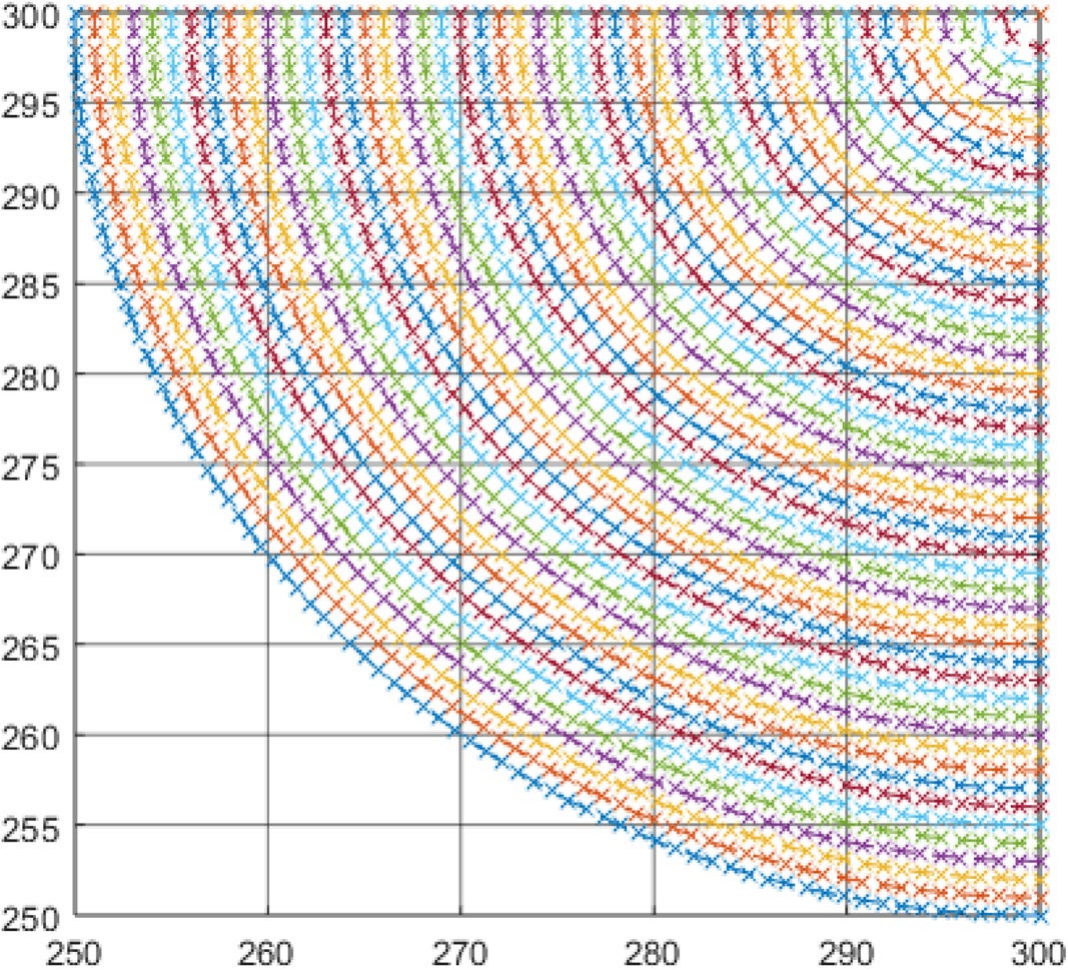
Quarter circle corners of a space point layer.

Equation (2) must be computed using the median (μ) and the standard deviation (σ) of the lightning current for both the positive and the negative lightning current^16,19^. The results from the use of the normal CDF and the approximated CDF using K_P2C_ will be compared for a cuboid structure, a cuboid structure with air termination and also for a free-standing and tall single rod air termination. Also, modifications to the rectangular space point layers will be applied to correct the superfluous space points by converting them to a quarter circle which perfectly fits into the collection volume of a cuboid structure, as illustrated in Fig. 6. This concept can be used to confine space point layers perfectly within the collection volume of the structure. The case of lightning strikes to a floating roof tank (FRT)^20^ is also considered in this study. These proposed modifications to the numerical DEGM will be implemented in MATLAB.

## Results

The results from the application of the improved model are presented in this section for the structural cases considered. The results obtained will be compared with published results where applicable.

### The results obtained using K_P2C_

**Figure 7:**
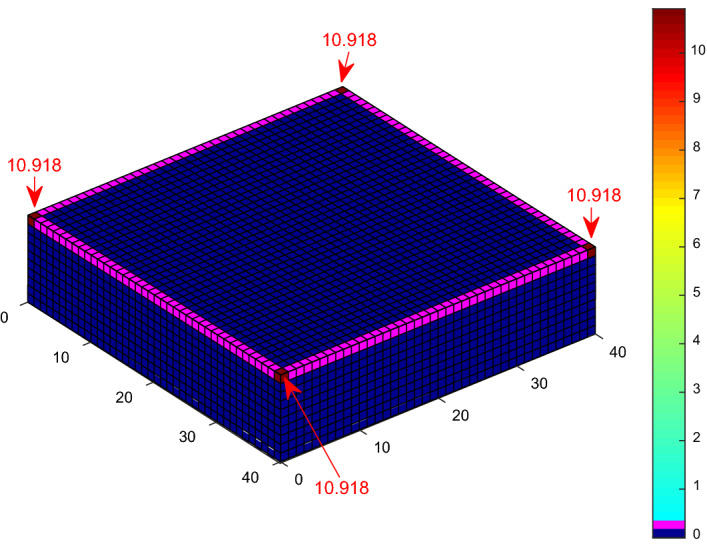
Lightning strike probability to a cuboid using K_P2C_.

The probability of a direct lightning strike to the meshed points of a 10 m high, 40 m × 40 m cuboid structure was evaluated using the CDF computation and approximated CDF using K_P2C_. The probability of a direct strike to each of the corners of the cuboid using an implementation of direct CDF and a 2 m × 2 m discretisation by Kern, et al.^21^ is 11.52%, while it is 11.197%, according to Adekitan and Rock^19^ using a discretisation of 1 m × 1 m. By applying K_P2C_ towards eliminating numerical errors with a discretisation of 1 m × 1 m, the probability of a direct strike to the corners is 10.918%, as shown in Fig. 7, which is very close to the analytical result of 10.77% obtained by Hannig, et al.^22^.

**Figure 8:**
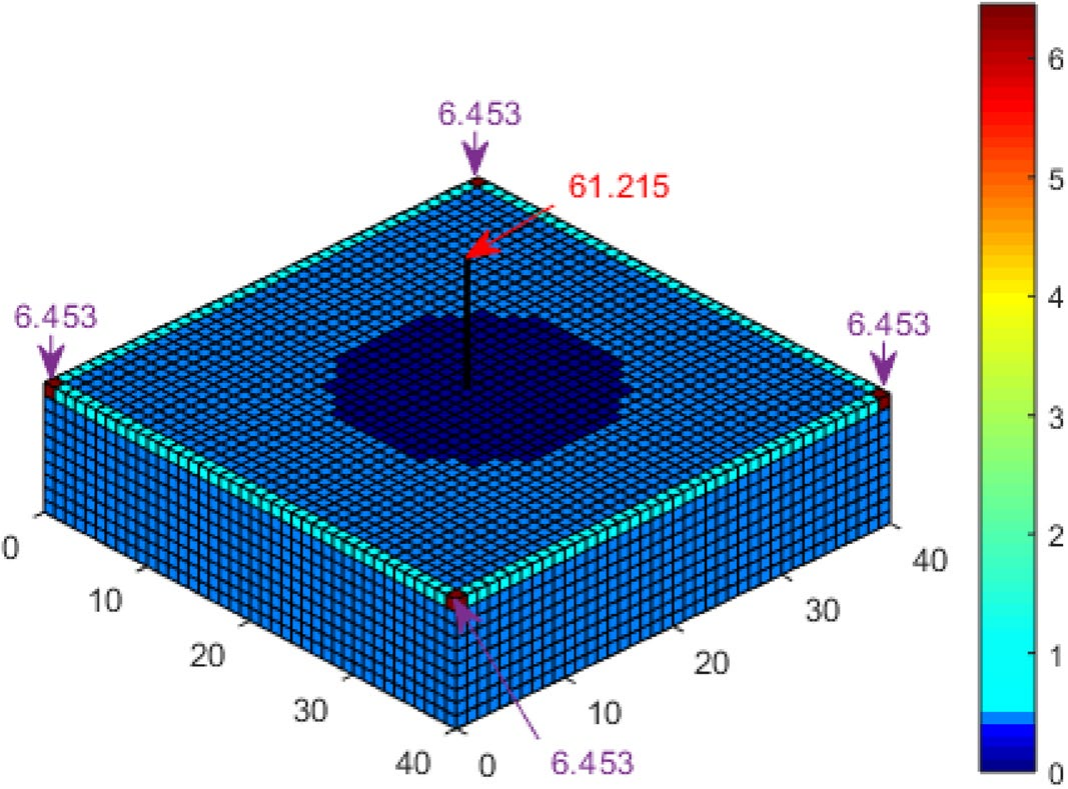
Lightning strike probability to a cuboid with central air termination using K_P2C_.

A second scenario in which the cuboid structure has a 10 m tall air termination installed at its centre was also considered. The probability of a direct strike to each of the corners of the cuboid, according to Kern, et al.^21^, is 6.15% and 64.95% for the tip of the air termination. In comparison, it is 6.334% for the corners and 63.154% for the air termination, according to Adekitan and Rock^19^ using a discretisation of 1 m × 1 m. By applying K_P2C_ with a discretisation of 1 m × 1 m, the probability of a direct strike to the corners is 6.453% and 61.215% for the air termination, as shown in Fig. 8, which is very close to the result of 61.49% for the air termination obtained by Hannig, et al.^23^ using an enhanced dynamic electro-geometrical model (eDEGM).

**Figure 9:**
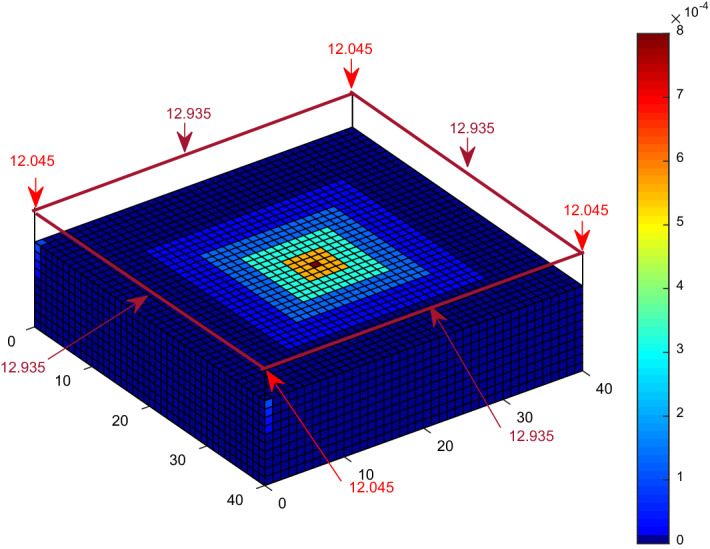
Lightning strike probability to a cuboid with catenary wires using K_P2C_.

A third scenario in which the cuboid structure has 4 m high catenary wires installed around the edges as air termination was also considered. The probability of a direct strike to each of the corners of the wires is 12.379% and 12.6095% for the inner section of the wires using the direct CDF computation. The probability of a direct strike to each of the corners of the wires with reduced numerical errors is 12.045% and 12.935% for the inner section of the wires using K_P2C_, as shown in Fig. 9.

**Figure 10:**
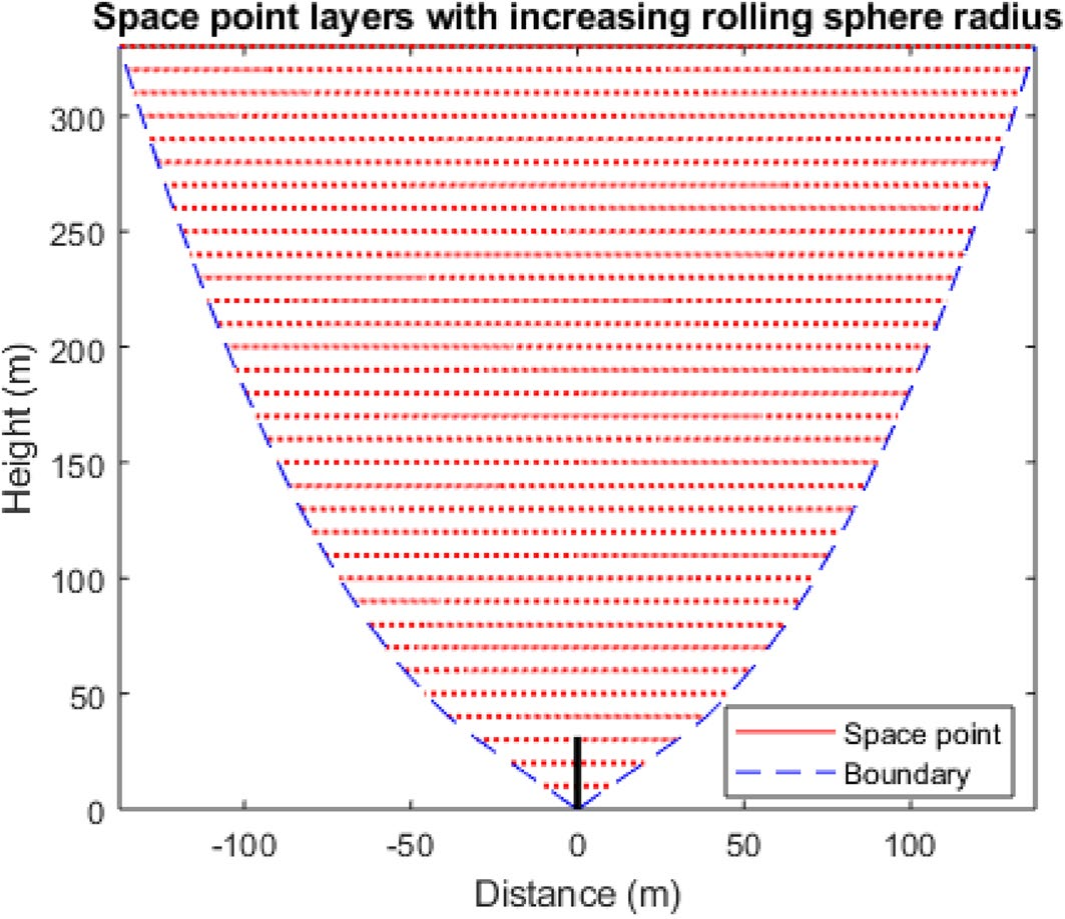
Space point layers and interception boundary around a 30 m high lightning rod.

A free-standing rod, as illustrated in Fig. 10, can be installed as an air termination to protect structures underneath its zone of protection. This study evaluates the probability of a lightning strike to four different heights of an air termination rod. Heights of 10 m, 30 m, 50 m, and 70 m were considered in the numerical analysis. The normal computation of the CDF was applied, and the improved model using K_P2C_. The results obtained are compared with published data using analytical techniques. In the model applied by Hannig, et al.^22^ for the computation of the strike, a lightning interception area of 1 km^2^ on flat ground was analysed around the structure. A 1 m^2^ area on flat ground has a cumulative probability of strike of 1, and as such, for 1 km^2^, the cumulative probability of a strike is 10^6^. This is true if all the surface of the ground is exposed to a direct strike, but in this case, the lightning rod offers a protective cover over a portion of the ground, and as such, the ground area within this protective cover has a reduced likelihood of a strike and none for some areas. This fact was not considered in the previous work. In this model, only the ground area outside the stretch of the interception boundary has a cumulative probability of strike of 1 per m^2^. For the ground area within the stretch of the interception boundary, the cumulative probability of each meshed point was computed. The summary of the result for a 1 km^2^ ground area using the assumed value of 10^6^ (G_1_) and the actual computed total cumulative probability of the ground (G_2_) are presented in Table 1, together with the published results. Table 1 presents the ratio of the total probability modulated collection volume (PMCV) of the rod to that of the ground.

**Table 1:** Comparison of the ratio of the lightning rod and ground PMCV.

	10 m	30 m	50 m	70 m
Published^22^	0.0067	0.0200	0.0330	0.0467
CDF (K_P2C_) G_1_	0.0066	0.0198	0.0331	0.0462
CDF (K_P2C_) G_2_	0.0066	0.0202	0.0341	0.0483
CDF G_1_	0.0071	0.0238	0.0452	0.0729
CDF G_2_	0.0071	0.0242	0.0466	0.0762

The results in Table 1 show a close approximation between the published data and the simulation result for the approximated CDF using K_P2C_. These results further confirm the improvement in the accuracy of the numerical DEGM by the use of the factor K_P2C_ in eliminating the effects of the numerical errors. A comparison between the results for G_1_ and G_2_ shows that the values are higher for G_2_ because of the reduced strike to the ground within the protective zone of the lightning rod.

### Elimination of ground surface points from DEGM computation

**Figure 11:**
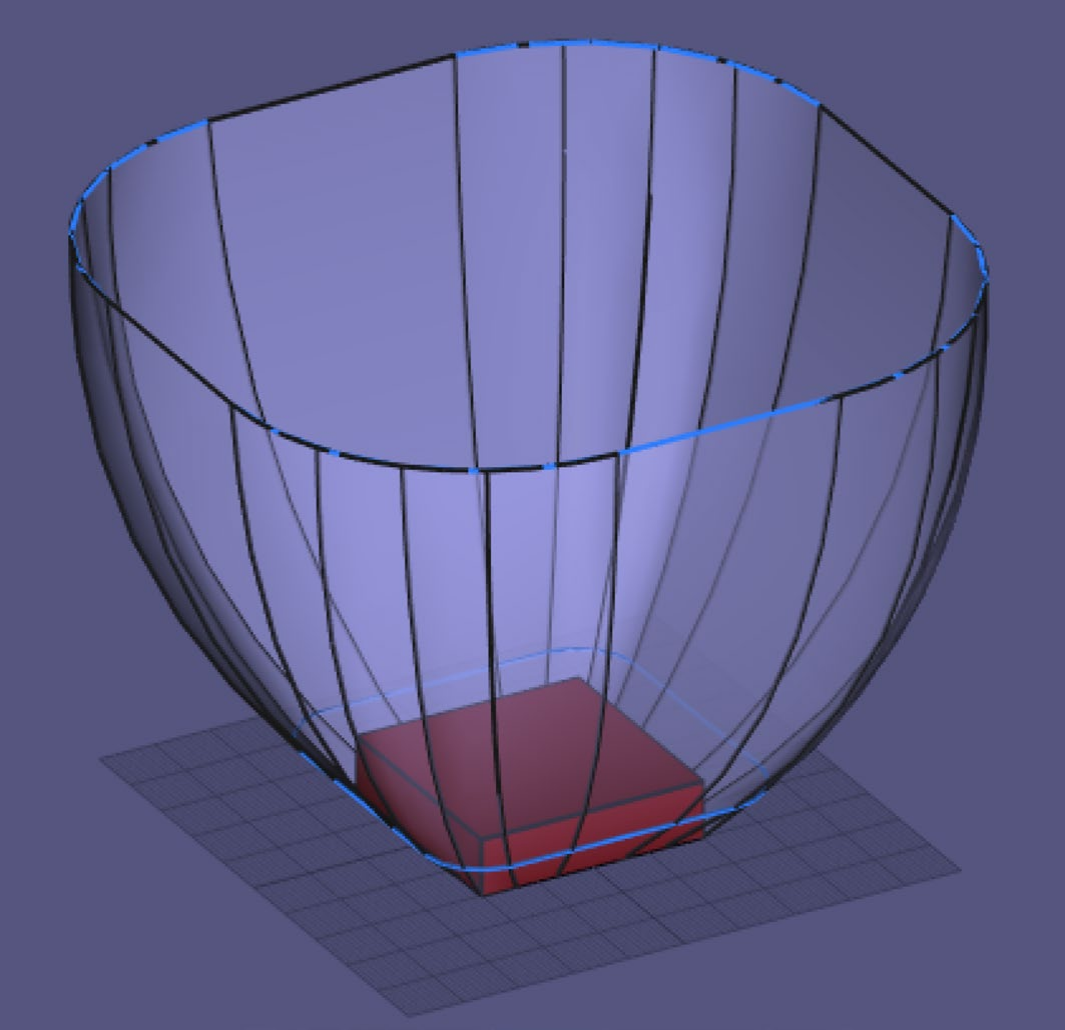
Collection volume around a cuboid with quarter-circle space point corners.

The use of rectangular space point layers bounded by the collection volume significantly reduces superfluous space points that are not needed in the computation of strikes within the collection volume. While this is effective, there is still a further opportunity to improve on the space point definition at corner points. As illustrated in Fig. 6, instead of a square-shaped space point area at the corners of each space layer for cuboid structure, it can be improved and converted to quarter circles that fit perfectly within the collection volume. The number of space points within the quarter circle corners should be approximately $\frac{\pi }{4}$ times the number of space points in the original square-shaped corners for any discretisation size. By implementing the quarter circle corners on the space point layers, instead of rectangular space point layers as shown in Fig. 4, the resulting collection volume is presented in Fig. 11, showing quarter-circle corners at the edge of the collection volume.

Modelling the space point layers with quarter-circle corners does not only eliminate the superfluous space points at the corners, but it has also confined all the space points perfectly within the collection volume of the cuboid, and this provides a major opportunity. In DEGM simulation, the point of a strike is determined by geometrical distance. For each space point, it must be determined whether a strike terminates on the structure or the ground, based on the closest in terms of distance, and as such, both the surface of this structure and nearby grounds must be meshed for analysis. Using a space point definition that is completely within the collection volume, i.e., all strikes from the collection volume are going to the structure, then there is no further need to include meshed ground-surface points in the modelling, and this will significantly reduce the computation time of DEGM simulations.

**Figure 12:**
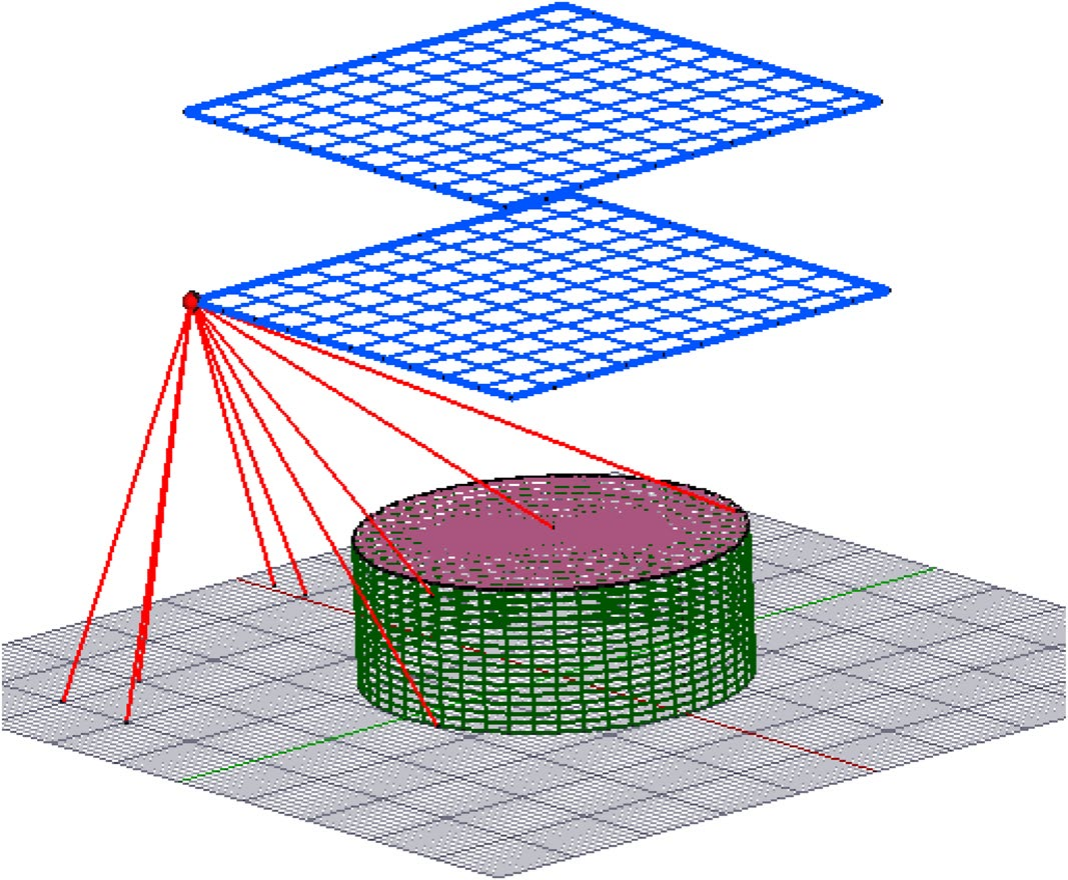
Space point layers above a FRT using rectangular space point layers.

**Figure 13:**
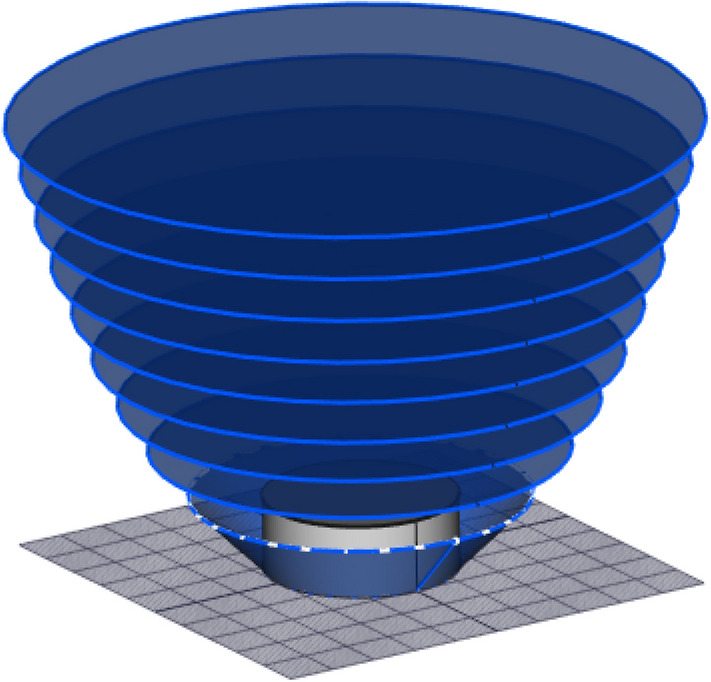
Space point layers confined within the collection volume of a FRT.

A similar concept can be applied to cylindrical tanks such as floating roof tanks (FRT). In place of rectangular space points, as illustrated in Fig. 12, which requires ground surface meshing, the space point layers can be modelled to fit perfectly within the collection volume of the floating roof tank, as shown in Fig. 13. These will eliminate iterations for the ground surface points and will ultimately improve the computation time of the model.

**Figure 14:**
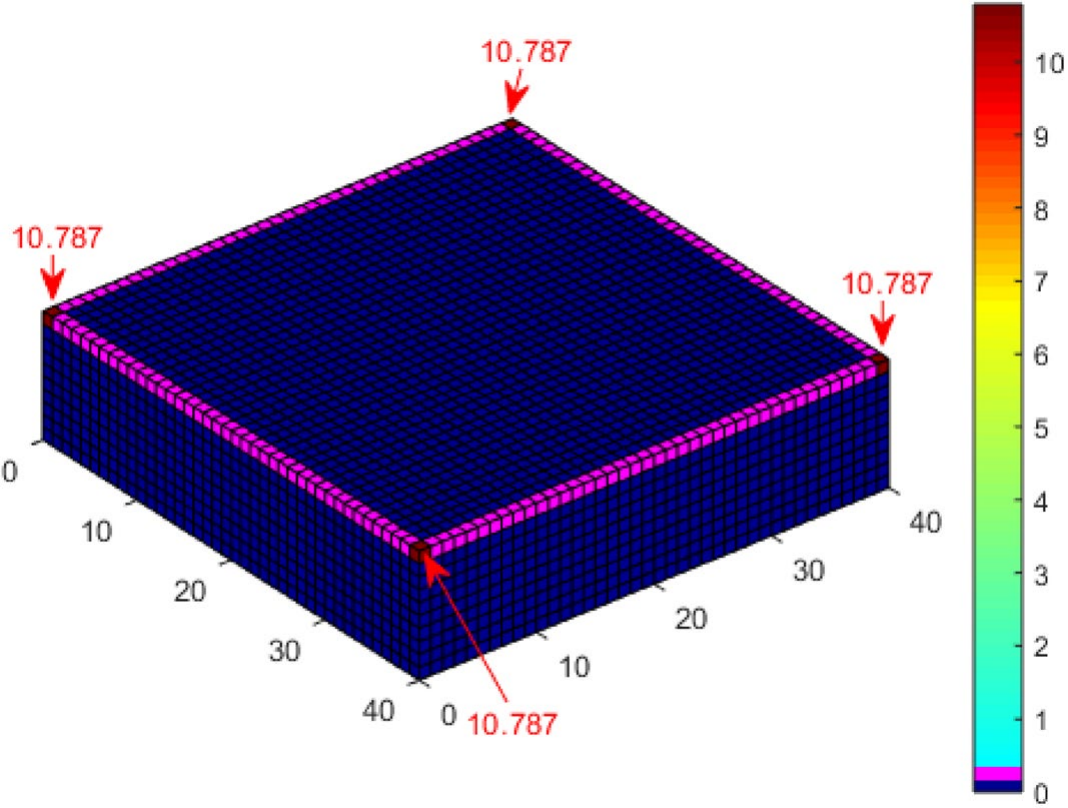
Lightning strike probability to a cuboid using K_P2C_ and confined space points.

The previous evaluation for a cuboid structure, a cuboid structure with a central air termination, and a cuboid with four catenary wires at the edges were repeated with space point definition completely within the collection volume of the cuboid and without ground surface points. The PDF to CDF conversion factor K_P2C_ was also applied in the simulation. The result for the cuboid structure is shown in Fig. 14. The probability of a direct strike to each of the corners is 10.787% as compared with 10.918% shown in Fig. 7. The result is approximately equal to the analytical result of 10.77% obtained by Hannig, et al.^22^.

**Figure 15:**
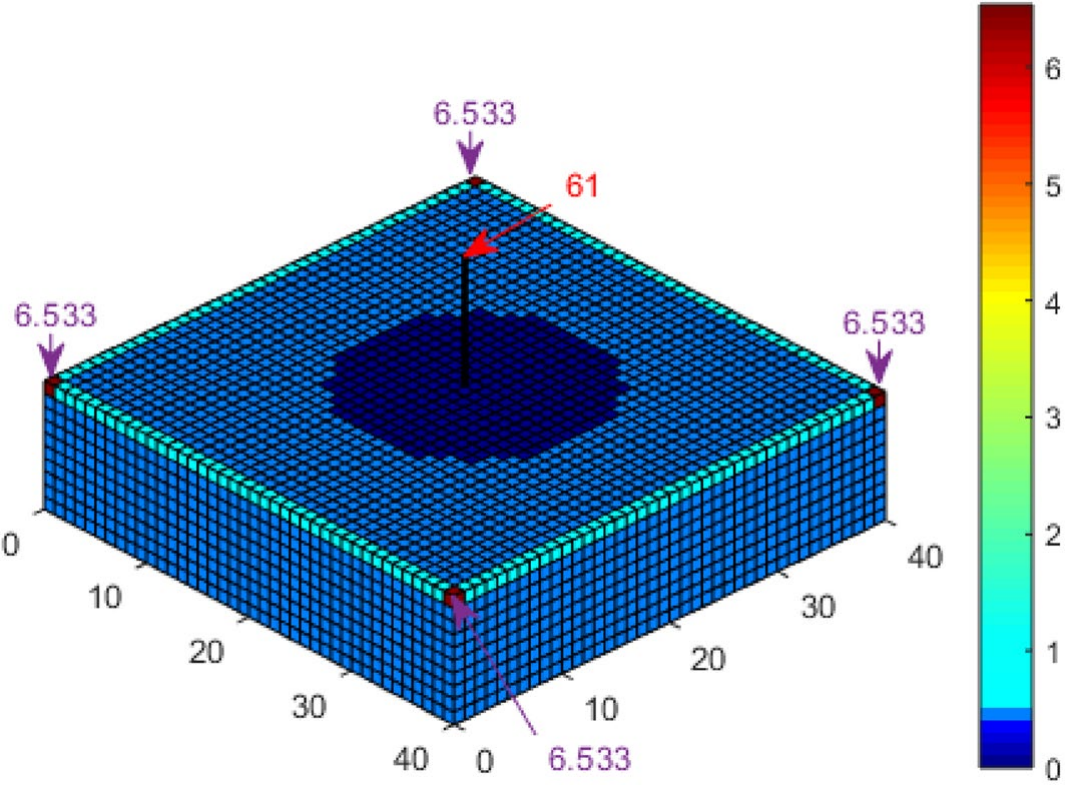
Lightning strike probability to a cuboid with central air termination using K_P2C_ and confined space points.

**Figure 16:**
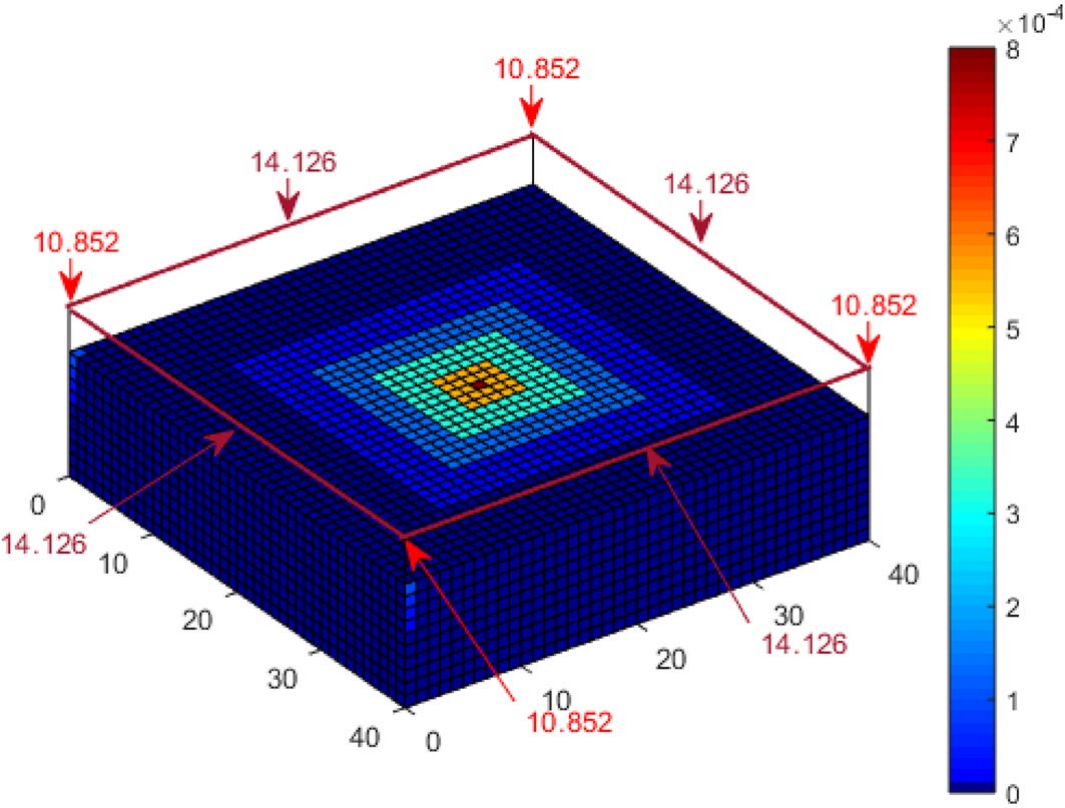
Lightning strike probability to a cuboid with catenary wires using K_P2C_ and confined space points.

For the cuboid with a central air termination, the probability of a direct strike to the corners, as shown in Fig. 15, is 6.533% and 61% for the air termination, as compared with 6.453% and 61.215% for the results shown in Fig. 8. Analysis by Hannig, et al.^23^ obtained 61.49% for the air termination. It is important to note that the collection volume of the central air termination alone is completely within the collection volume of the cuboid structure. Figure 16 presents the result for the four catenary air termination wires. In Fig. 9, the overall probability of a direct strike to the four wires is 99.92%, while in Fig. 16, it is 99.91%. These values are approximately equal, but based on the distribution of the percentages, there is a significant difference between the two results. In Fig. 9, for the rectangular space point layers, the probability of a direct strike to each of the four corners of the catenary wires is 12.045% and 12.935% for the remaining section of each wire, whereas it is 10.852% and 14.126% using the confined space point layers. Now the question, why is there a significant difference in the result? This result is not only interesting, but it also highlights a reality that could be easily missed. For the rectangular space point layers, there are superfluous space points that are not within the natural collection volume of the cuboid but are within the collection volume of the air termination because of its height. The lightning rod intercepted the strikes from these extra space points when the rectangular space points layer was applied. For the confined space points which are completely within the collection volume of the cuboid, these extraneous space points no longer exist. As such, there is a redistribution of the strike-interception percentages. This does not necessarily translate to the fact that air terminations attract more lightning to the structure. It only implies that with the higher height of air terminations above a structure, especially when positioned at the edge of a structure, the air terminations can safely intercept lightning leaders beyond the collection volume of the protected structure. It may therefore be important when applying DEGM to state whether the focus of the analysis is within the collection volume of the structure or not. For analysis within the collection volume of the structure, the percentage probability of strike to air terminations indicates the percentage of the strikes within the volume captured by the air terminations. When analysis goes beyond the collection volume of the structure, then the percentage of a strike to the air terminations includes strikes from outside the collection volume of the structure, which will not necessarily terminate directly on the structure in terms of geometric distances.

**Figure 17:**
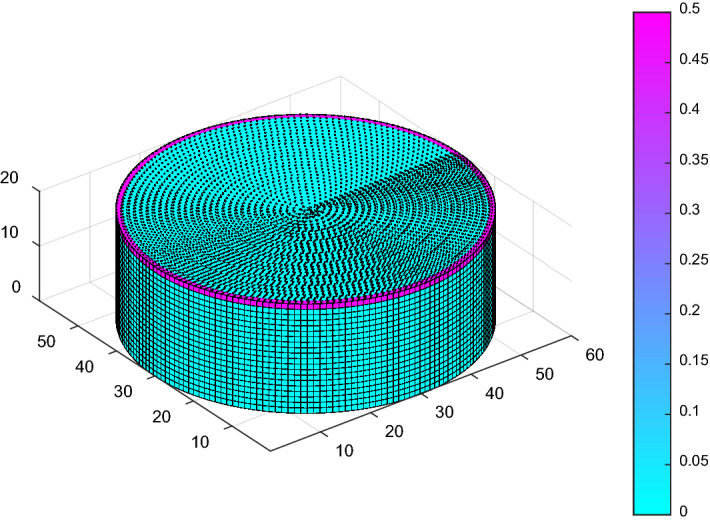
Lightning strike probability to a FRT of 60 m in diameter.

A DEGM simulation can be applied to any structure, provided its surface can be effectively modelled as a mesh. DEGM was also implemented for a FRT of 60 m in diameter and 20 m in height with its roof at the apex height. The floating roof tank is susceptible to lightning-induced fires, which may result in prolonged fires and explosions with an extensive impact on the environment^24,25^. The percentage probability of a lightning strike to the meshed points on the FRT is presented in Fig. 17. Using rectangular space point layers within the collection volume corresponding to Fig. 12, the summed probability of a direct strike to the rim edge of the FRT is 90.305%, 0.044% for the sidewall, and 9.651% for the floating roof. Using the circular spaced-point layer corresponding to Fig. 13, the summed probability of a strike to the rim edge of the FRT is 90.586%, 0.052% for the sidewall, and 9.362% for the floating roof. The accuracy of the results confirms the suitability of circular space point layer definition for a FRT with the advantage of a significant reduction in computation time.

The major advantage of limiting the space points within the collection volume of the structure is to reduce the number of iterations needed to generate the result. Table 2 presents the computation time on MATLAB running on windows 10 for the FRT and cuboid cases considered. The computation times of three space point definitions are presented. These are linearly increasing space point layers with ground surface points according to Adekitan and Rock^19^, rectangular space point layers around the collection volume with ground surface points as illustrated in Fig. 4. The third approach uses space point layers completely confined to the collection volume of the structure, as illustrated in Fig. 11 for the cuboid structure. The computation time comparison gives a perspective of the extent of the impact of space point definition on the computation time of strike probabilities using numerical simulations.

**Table 2:** DEGM computation time comparison.

	Linearly increasing space point (h)	Collection volume with ground (h)	Collection volume without ground (min)
Cuboid	86.8	14	5.75
Cuboid and central air terminal	114.7	13.7	5.64
Cuboid and catenary wires	115.62	13.51	5.9
FRT	113.28	27.38	28.8

### Interpretation of the numerical results

The numerical DEGM simulations for a cuboid structure without any LPS show that the corner points with a strike probability of 10.918% using K_P2C_ are the most likely strike points. For the cuboid structure with a central air termination, the interception efficiency is 61% as compared to 99.91% using four catenary wires. This shows the inefficacy of poor air termination design and positioning. Air terminals of the appropriate type and numbers must be installed at high-risk points to ensure adequate protection. The DEGM simulation for the FRT shows that the rim edge has the highest likelihood of a direct strike of 90.586%. The result in Table 2 shows a drastic change in the computation time from several hours to a few minutes just by changing the definition of the space point layers without impacting the overall accuracy of the results obtained. Reducing the space points needed in the computation allowed the removal of ground surface points and, ultimately, the number of required iterations. A major time advantage can now be achieved in DEGM implementation.

As discussed in this study, the proposed modifications to the modelling of DEGM have significant advantages in terms of improved accuracy and reduced computation time. The applications of the improved model focused on a cuboid and a cylindrical FRT. While these two structures have a simple geometrical configuration, implementing the modifications to the space definitions on MATLAB requires tricky iterative code implementation. Implementing the same on complex structures with complex-shaped collection volumes may be difficult to achieve.

## Conclusion

The lightning hazard is a reality that has to be adequately managed to protect lives and properties. Lightning protection requires detailed compliance with established guidelines and procedures for implementing efficient and effective protection for structures and facilities. The number, position and arrangement of air terminations are vital attributes that determine the ability of a lightning protection system to intercept downward leaders safely. Air terminations must be positioned at high-risk points on a structure. It is therefore vital to be able to identify high strike-risk locations on a structure. The dynamic electro-geometrical model is a tool that enables the characterisation of the surface of a structure in terms of the likelihood of a direct strike. The numerical implementation of the dynamic electro-geometrical model has associated numerical errors such as the discretisation size effect, which impacts its accuracy, and also, it is computationally intensive to implement. This study identified sources of numerical errors and proposed modifications to the definition of space point layers toward reducing the computation time and improving the accuracy of the dynamic electro-geometrical model simulations.

A probability density function to cumulative distribution function conversion factor called K_P2C_ was developed and applied in the computation of the strike probability, and this helped to eliminate the effects of the numerical errors. For the cuboid structure that was evaluated within its collection volume, a reduction in computation time from 14 h to 5.75 min was achieved by eliminating the ground surface points. This shows a significant computation time advantage. The results obtained for the cuboid structure with catenary wires emphasise the need to state when applying DEGM to structures with air terminations whether the focus of the analysis is within the collection volume of the structure or not. The improved dynamic electro-geometrical model (IDEGM) proposed in this study has successfully reduced the computation time from more than several hours to less than 30 min for all the cases considered.
