# Publisher Correction: Improving the computational accuracy of the dynamic electro-geometrical model using numerical solutions

**DOI:** 10.1038/s41598-022-10832-6

**Published:** 2022-04-20

**Authors:** Aderibigbe Israel Adekitan

**Affiliations:** Group for Lightning and Surge Protection, Technische Universität Ilmenau, Ilmenau, Germany

Correction to: *Scientific Reports* 10.1038/s41598-022-09674-z, Published online 06 April 2022

The original version of this Article contained a typographical error in Table 2, where the unit of the computation time for the “Collection volume without ground” was incorrect.

“minh)”

now reads:

“(min)”

The original article has been corrected.

